# Correlation of Lymphocyte‐to‐C‐Reactive Protein Ratio With Erectile Dysfunction Risk in United States Adult Males during 2001–2004: A Cross‐Sectional Analysis

**DOI:** 10.1002/hsr2.71585

**Published:** 2025-11-26

**Authors:** Junjie Ni, Cong Jin, Chaoyang Xu, Shunlan Ni, Lin Lv

**Affiliations:** ^1^ Department of Breast and Thyroid Surgery, Affiliated Jinhua Hospital Zhejiang University School of Medicine Jinhua Zhejiang Province China; ^2^ Department of infectious disease, Affiliated Jinhua Hospital Zhejiang University School of Medicine Jinhua Zhejiang Province China

**Keywords:** Cross‐sectional study, Erectile dysfunction, lymphocyte‐to‐C‐reactive protein ratio, NHANES, United States adults

## Abstract

**Background and Aims:**

Erectile dysfunction (ED) is a common condition in aging males and may be influenced by systemic inflammation. This study aimed to examine the association between the lymphocyte‐to‐C‐reactive protein ratio (LCR), a novel inflammatory marker, and ED prevalence in the adult population of the United States.

**Methods:**

Data were obtained from the 2001–2004 National Health and Nutrition Examination Survey (NHANES). Male participants aged ≥ 20 years with available LCR and ED data were included. Weighted univariate and multivariable logistic regression analyses were performed to assess the association between LCR and ED. Additional analyses included subgroup assessments, interaction testing, and restricted cubic spline modeling to evaluate nonlinearity.

**Results:**

A total of 3952 male participants were included, with a mean LCR of 2.52 ± 0.08 and an ED prevalence of 19.47%. Higher LCR quartiles were associated with a lower prevalence of ED. After adjusting for potential confounders, individuals in the highest LCR quartile had a 37% lower risk of ED compared to those in the lowest quartile (OR: 0.63, 95% CI: 0.41–0.95; *p* = 0.03). Two‐piece‐wise regression identified an LCR threshold at 3.67. Below this value, LCR was inversely associated with ED risk (OR = 0.70; *p* = 0.01); above it, no significant association was observed (*p* = 0.88). Subgroup and interaction analyses confirmed the robustness of the association across different populations.

**Conclusions:**

LCR is a reliable, readily measurable biomarker that is independently associated with reduced ED risk. It may serve as a promising inflammatory marker for identifying individuals at lower risk of ED.

## 1 Introduction

Erectile dysfunction (ED) is quite common in men, and it progressively impacts a larger male population with age [1]. Before 40 years of age, ED incidence ranges between 1% and 10%. Post 40 years of age, ED incidences increase drastically to 52% [2], with an alarming 70% occurring among ≥ 70 year old men [3]. According to Ayta IA, ED incidence exhibit an increasing trend, with a projected > 322 million global male sufferers by 2025 [4]. ED diagnosis is rather elusive as it is reliant upon patient reporting. Thus, there is great potential for erroneous intervention, which may exacerbate the condition and introduce substantial financial strain. Not to mention, with a rise in ED incidences, the socioeconomic burden becomes substantial, with preventative and intervention‐based expenditure surpassing $15 billion [4], with added hidden expenses. Despite its nonlife threatening nature, ED can have significant negative consequences on relationships, mood, and patient quality of life [5]. ED etiology is multi‐faceted, and may involve processes, such as, vascular, hormonal, neurological, and anatomical factors [6]. Appropriate identification of ED risk factors (RFs), namely, smoking, obesity, sedentary lifestyle, and persistent alcohol intake, is critical to ED prevention [7].

Emerging reports suggested that ED development and severity are closely associated with inflammatory and endothelial dysfunction markers [8]. Complete blood counts are routine and cost‐efficient evaluations that can potentially estimate inflammation. The lymphocyte‐to‐C‐reactive (LCR) protein ratio is one such inflammatory biomarker with strong promise as a prognostic and diagnostic indicator for multiple diseases [9, 10, 11]. LCR refers to the ratio between absolute lymphocyte quantity and C reactive protein (CRP) content. More recently, it was demonstrated that LCR can predict disease outcomes and guide clinical decision‐making in several situations, such as, cancer, cardiovascular disease, and COVID‐19 [9, 10, 11, 12]. Hence, LCR is a robust predictive marker for inflammation and disease prognosis. However, till date, no studies have evaluated the potential link between LCR and ED.

In this report, we analyzed a relatively large patient population, with comprehensive data (and confounder adjustment), to examine the potential link between LCR and ED. We speculated a strong association between the two factors. Our findings may enhance the current mechanistic understanding of the ED‐based inflammatory activities, and pave way for robust diagnostic marker identification for this condition.

## 2 Materials and Methods

### 2.1 Study Participants and Data Collection

**Figure 1:**
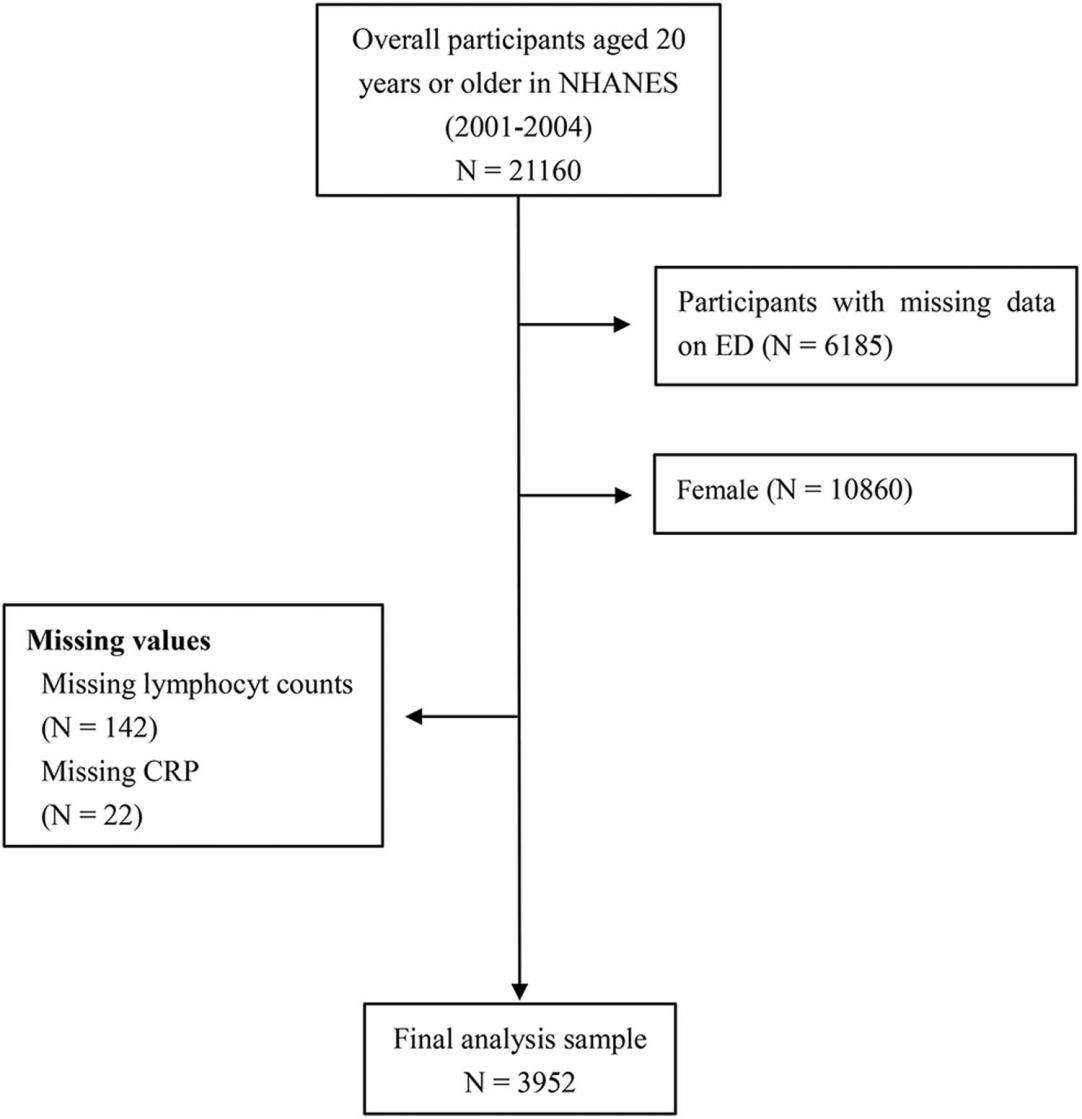
Illustration of the research design. Abbreviations: RP, C reactive protein; NHANES, National Health and Nutrition Examination Survey.

This study followed the principles of the CONSORT guidelines, where applicable to observational data derived from a population‐based cross‐sectional survey. The manuscript was structured to ensure transparent reporting of participant selection, variable definitions, and analytical procedures to enhance reproducibility and comparability across studies. We downloaded data from 2001 to 2004 NHANES, an extensive, multi‐step, probability‐clustering method of collecting health and nutritional data of US adults and children. NHANES employed oversampling of different ethnicities, namely, Non‐Hispanic black (NHB), Non‐Hispanic white (NHW), Mexican American (MA), among others. The raw data set included 21,161 over the age of 20. Participants were eligible for inclusion if they were male, aged 20 years or older, and had complete data available for ED status, lymphocyte count, and CRP level. Individuals were excluded if they were female, younger than 20 years, or had missing data on ED, lymphocyte count, CRP, or any covariates required for multivariable adjustment. However, for our assessment, we excluded females (*n* = 10,860), as well as those with unavailable ED (*n* = 6185), lymphocyte count (*n* = 142), and CRP data (*n* = 22). Following application of our rigorous inclusion and exclusion criteria, we gathered information on approximately 3952 subjects (Figure 1). A detailed participant flow diagram is provided in Figure 1, conforming to CONSORT flowchart recommendations. This diagram visually represents the number of participants included and excluded at each stage of analysis, including reasons for exclusion. The NHANES protocol was approved (Protocol #2001–2004, the website is https://www.cdc.gov/nchs/nhanes/about/erb.html) by the National Center for Health Statistics Ethics Review Board, with participant informed consent before study initiation [13]. Therefore, there was no need for external ethical and informed consent endorsement. All data analyses strictly followed the NHANES recommendations and regulations, and Authors had no access to information that could identify individual participants [14]. Our secondary analysis adheres to the Strengthening the Reporting of Observational Studies in Epidemiology (STROBE guidelines) for cohort studies. Comprehensive details about the NHANES survey can be accessed publicly at https://wwwn.cdc.gov/nchs/nhanes.

### 2.2 Exposure Variables and Outcomes

Herein, the lymphocyte counts and CRP level can be obtained directly from laboratory data files. The LCR represented the exposure variable. To adjust for variations in LCR values, we adopted LCR transformation. LCR was computed by dividing the lymphocyte counts by CRP content and then dividing the quotient by 10 [15].

ED (KIQ400) assessment was based on a well‐established questionnaire. The questionnaire included inquiries like, “Please describe your ability to achieve and sustain an erection adequate for sexual intercourse.” Participants were allowed to respond either by selecting “never,” “sometimes,” “usually,” and “almost always or nearly always.” ED instances were described as “sometimes able” or “never able,” and was our main measurement endpoint [7]. ED prevalence served as the endpoint of interest. Definitions of exposure and outcome variables were established a priori before analysis. The primary outcome was ED prevalence, based on a validated NHANES questionnaire item. The main exposure was the LCR, treated both as a continuous and categorical variable.

### 2.3 Biochemical Parameters

We conducted routine circulating biochemical evaluation [16, 17], using blood from the volunteers mobile examination centers (MECs). Among the analyzed variables were estimated glomerular filtration rate (eGFR), high‐density lipoprotein cholesterol (HDL‐C) and fasting total cholesterol (TC). For renal function assessment, eGFR was computed via the Chronic Kidney Disease Epidemiology Collaboration (CKD‐EPI) equation and utilized creatinine content, given by: eGFR = 141 × min(Scr/κ, 1)^α^ × max(Scr/κ, 1)^−1.209^ × 0.993^Age^ × 1.018 [if female] _ 1.159 [if black], whereby Scr represents circulating creatinine, κ represents 0.7 (females) and 0.9 (males), α represents −0.329 (females) and −0.411 (males), min represents Scr/κ minimum or 1, and max represents Scr/κ maximum or 1 [18].

### 2.4 Other Covariates of Interest

Herein, we explored several possible ED‐associated confounding factors, screened based on epidemiological investigations. Among the assessed socioeconomic variables were age, race, educational status, marital status, and family poverty‐to‐income ratio (PIR), while the physical parameter was body mass index (BMI), lifestyle parameters were alcohol intake and smoking, and comorbid parameters were diabetes mellitus (DM), hypertension, hyperlipidemia, CKD, cardiovascular disease (CVD) and asthma.

Sociodemographic variables (self‐reports) were classified as follows: age, < 50, or ≥ 50 years; race/ethnicity, NHB, NHW, MA, or others, covering a broad range of racial backgrounds, as well as examination of marital status, categorizing cases as either never married, currently married, or living separately. The latter classification denoted those who were either divorced, widowed, or residing in distinct households; educational status, less educated than high school (HS), HS graduate, or beyond HS; BMI ≥ 25 kg/m^2^ were considered overweight, including obese (World Health Organization (WHO) standards); PIR, < 1.00 represented below poverty cut‐off, PIR > 3.00 meant 3× above poverty cut‐off.

Lifestyle variables (self‐reports) were classified as follows: alcohol intake, those who had had < 12 drinks in lifetime, those who had had ≥ 12 drinks in their lifetime but were not currently drinking, those taking ≤ 3 drinks per week, and those taking > 3 drinks per week; smoking habit (according to NCHS and CDC guidelines) included participants who had never smoked or smoked < 100 cigarettes in their lifetime, those who had smoked ≥ 100 cigarettes but were not currently smoking, and current smokers.

Subject comorbidities and corresponding definitions are as follows: hypertension, diagnosed with hypertension, using anti‐hypertensive medication, or systolic blood pressure (BP) ≥ 140 mmHg and/or diastolic BP ≥ 90 mmHg; type 2 DM, diagnosed with the disease, oral glucose tolerance ≥ 11.1 mmol/L, random glucose ≥ 11.1 mmol/L, fasting glucose ≥ 7.0 mmol/L, hemoglobin A1c (HbA1c) ≥ 6.5%, or using antidiabetic medication; CKD, eGFR was computed based on the CKD‐EPI formula, eGFR < 60 ml/min/1.73 m^2^, or urine albumin‐to‐creatinine ratio (UACR) > 30 mg/g [19]; hyperlipidemia was described as HDL‐C content < 40 mg/dL, LDL‐C content ≥ 160 mg/dL, triglycerides ≥ 200 mg/dL, and TC ≥ 240 mg/dL, or via a prior diagnosis made during the NHANES blood examination. The presence of CVD and asthma was determined via self‐report with subject responding ‘Yes’ or ‘No’. All covariates were identified based on previous literature and included to control for potential confounding effects. Definitions for comorbidities and lifestyle factors followed NHANES standard protocols, ensuring uniform classification across all participants.

### 2.5 Statistical Analysis

The statistical analysis was conducted using R software (version 4.2.0; R Foundation for Statistical Computing, Vienna, Austria). Statistical reporting adhered to the Guidelines for Reporting of Statistics for Clinical Research in Urology [20] and the Statistical Analyses and Methods in the Published Literature (SAMPL) guidelines, with an emphasis on transparent model specification, effect size interpretation, and appropriate handling of missing data.

Survey weights provided by NHANES were applied to account for its complex, stratified, multistage probability cluster sampling design, minimizing bias from oversampling in specific demographic groups [21]. For descriptive statistics, survey‐weighted means and standard errors (SEs) were reported for continuous variables, and survey‐weighted proportions for categorical variables. Comparisons between groups (e.g., across LCR quartiles and ED status) were conducted using survey‐weighted chi‐square tests for categorical variables and survey‐weighted t‐tests for continuous variables.

To examine the association between LCR and ED, we developed both unadjusted and multivariable survey‐weighted logistic regression models. LCR was analyzed both as a continuous variable and as a categorical variable stratified by quartiles. Multivariable models were pre‐specified to minimize the risk of overfitting. The crude model included no covariate adjustment. Model 1 adjusted for age alone, and Model 2 included adjustments for age, BMI, marital status, PIR, education level, alcohol consumption, smoking status, hypertension, hyperlipidemia, CKD, CVD, diabetes, asthma, and eGFR.

Effect sizes were presented as odds ratios (ORs) with 95% confidence intervals (CIs). All hypothesis tests were two‐sided, and a *p*‐value < 0.05 was considered statistically significant. The linearity of the LCR–ED relationship was assessed using restricted cubic spline (RCS) regression with three knots, placed at percentiles determined by minimizing the Akaike Information Criterion (AIC). Threshold effects were evaluated using segmented (piecewise) regression models, and model fit comparisons between linear and nonlinear forms were assessed using the likelihood ratio test (LRT).

Model diagnostics were performed where applicable, including assessment of multicollinearity, testing for interaction terms, and identification of influential observations. Missing data were handled using complete‐case analysis, as the proportion of missingness was < 5% and assumed to be missing completely at random (MCAR). All statistical terms, symbols, and abbreviations were defined at first use and applied consistently.

All statistical tests were two‐sided, and a priori significance was defined at *p* < 0.05. Model specifications, variable selection, and subgroup analyses were pre‐specified unless otherwise stated. No imputation was performed, as missing data were minimal (< 5%) and assumed to be MCAR. The analysis strategy was designed and conducted according to established epidemiological and statistical principles to allow replication and facilitate meta‐analytic inclusion in future reviews. This observational analysis adheres to the relevant items from the STROBE extension of CONSORT, tailored for reporting cross‐sectional studies.

## 3 Results

### 3.1 Personal Profiles of Participants

Totally, 3952 participants were enrolled, with an average age of 45.17 ± 0.38 years, average LCR index of 2.52 ± 0.08, and a mean overall ED incidence of 19.47%. ED prevalence decreased with increasing LCR quartiles (Quartile 1: 31.64%, Quartile 2: 20.34%, Quartile 3: 17.72%, Quartile 4: 10.64%). Among the four LCR quartiles, with the exception of asthma (*p* = 0.13, Chi‐square test), the distributions of age, ethnicity, marital status, educational status, family PIR, smoking, drinking, BMI, HDL‐C, TC, eGFR, DM, hypertension, hyperlipidemia, CKD, and CVD showed significant differences across quartiles (all *p* < 0.05, Chi‐square or weighted *t*‐tests as appropriate). Compared to the lowest LCR quartile, subjects in the highest LCR quartile had significantly higher HDL‐C and eGFR levels (*p* < 0.001 for both, weighted *t*‐tests), were more likely to have education above high school, and less likely to have BMI ≥ 25 kg/m², DM, hypertension, hyperlipidemia, CKD, and CVD (all *p* < 0.01, Chi‐square test). Additionally, participants in the lowest LCR quartile were older, more likely to be Non‐Hispanic Black, living separately, to have less than high school education, and to be former or current smokers and former drinkers (all *p* < 0.01, Chi‐square test) (Table 1).

**Table 1:** Baseline characteristics of the study population.

Characteristics (weighted)	LCR categories					
	Total (*N* = 3952)	Q1(≤ 0.52)	Q2 (> 0.52, ≤ 1.43)	Q3 (> 1.43, ≤ 2.6)	Q4 (> 2.6)	*p*‐value
TC (mmol/L) (Mean ± SE)	5.18 ± 0.02	5.18 ± 0.06	5.34 ± 0.05	5.25 ± 0.03	4.98 ± 0.04	< 0.0001
HDL‐C (mmol/L) (Mean ± SE)	1.22 ± 0.01	1.15 ± 0.01	1.17 ± 0.01	1.22 ± 0.01	1.31 ± 0.02	< 0.0001
eGFR (ml/min/1.73m^2^) (Mean ± SE)	93.14 ± 0.55	89.23 ± 0.76	91.99 ± 0.72	92.99 ± 0.83	97.47 ± 0.71	< 0.0001
Erectile dysfunction, *n* (%)						< 0.0001
No	2794 (80.53)	566 (68.36)	696 (79.66)	723 (82.28)	809 (89.36)	
Yes	1158 (19.47)	426 (31.64)	293 (20.34)	276 (17.72)	163 (10.64)	
Age (years), *n* (%)						< 0.0001
< 50	2036 (63.01)	379 (52.04)	454 (57.67)	533 (64.81)	670 (74.88)	
≥ 50	1916 (36.99)	613 (47.96)	535 (42.33)	466 (35.19)	302 (25.12)	
Race, *n* (%)						0.05
Mexican American	812 (7.81)	164 (6.06)	205 (6.89)	221 (8.14)	222 (9.75)	
Non‐Hispanic Black	720 (9.32)	214 (11.53)	170 (9.04)	157 (8.15)	179 (8.92)	
Non‐Hispanic White	2162 (74.38)	568 (75.38)	546 (74.97)	551 (75.46)	497 (72.02)	
Other race	258 (8.49)	46 (7.04)	68 (9.10)	70 (8.26)	74 (9.31)	
Marital status, *n* (%)						< 0.0001
Married	2720 (69.91)	657 (67.03)	711 (72.71)	730 (74.64)	622 (65.11)	
Live separated	550 (11.54)	194 (16.88)	140 (11.15)	128 (11.03)	88 (8.10)	
Never married	680 (18.44)	141 (16.09)	136 (15.69)	141 (14.33)	262 (26.78)	
Missing	2 (0.11)	0 (0.00)	2 (0.45)	0 (0.00)	0 (0.00)	
Education level, *n* (%)						0.04
Less than high school	533 (6.02)	170 (8.29)	131 (5.89)	132 (5.70)	100 (4.66)	
High school	1556 (37.92)	400 (41.22)	382 (36.83)	378 (36.83)	396 (37.34)	
More than high school	1861 (55.99)	422 (50.49)	475 (57.23)	489 (57.47)	475 (57.82)	
Missing	2 (0.06)	0 (0.00)	1 (0.05)	0 (0.00)	1 (0.19)	
Family PIR, *n* (%)						0.01
< 1	553 (9.83)	151 (12.21)	113 (8.03)	141 (10.20)	148 (11.05)	
1–3	1548 (32.13)	426 (38.75)	388 (32.36)	372 (31.45)	362 (33.38)	
> 3	1645 (53.15)	364 (49.04)	432 (59.61)	443 (58.35)	406 (55.57)	
BMI (kg/m^2^), *n* (%)						< 0.0001
< 25	1144 (29.04)	197 (19.51)	198 (18.86)	283 (27.84)	466 (48.50)	
≥ 25	2711 (69.46)	753 (80.49)	772 (81.14)	690 (72.16)	496 (51.50)	
Smoking status, *n* (%)						0.002
Never	1587 (42.66)	326 (34.54)	396 (41.53)	413 (45.20)	452 (47.74)	
Former	1297 (29.13)	379 (33.37)	327 (28.99)	332 (28.75)	259 (26.24)	
Now	1064 (28.18)	287 (32.09)	266 (29.48)	252 (25.98)	259 (25.98)	
Missing	4 (0.03)	0 (0.00)	0 (0.00)	2 (0.08)	2 (0.04)	
Alcohol usage, *n* (%)						0.05
Never	285 (6.98)	74 (6.72)	66 (6.40)	67 (6.58)	78 (8.08)	
Former	817 (16.73)	258 (20.99)	216 (17.75)	198 (16.51)	145 (12.59)	
Moderate	63 (1.93)	14 (2.13)	21 (3.05)	15 (1.35)	13 (1.33)	
Heavy	944 (25.33)	215 (23.21)	222 (24.20)	234 (25.48)	273 (27.91)	
Missing	1843 (49.04)	431 (46.94)	464 (48.60)	485 (50.08)	463 (50.09)	
DM, *n* (%)						< 0.0001
No	3380 (89.53)	777 (82.83)	829 (87.44)	872 (91.13)	902 (95.26)	
Yes	572 (10.47)	215 (17.17)	160 (12.56)	127 (8.87)	70 (4.74)	
Hypertension, *n* (%)						< 0.0001
No	2466 (68.15)	492 (56.28)	586 (64.62)	646 (68.91)	742 (80.13)	
Yes	1483 (31.72)	500 (43.72)	403 (35.38)	352 (30.88)	228 (19.61)	
Missing	3 (0.13)	0 (0.00)	0 (0.00)	1 (0.21)	2 (0.26)	
Hyperlipidemia, *n* (%)						< 0.0001
No	1086 (27.97)	207 (20.97)	217 (21.13)	261 (26.59)	401 (41.14)	
Yes	2866 (72.03)	785 (79.03)	772 (78.87)	738 (73.41)	571 (58.86)	
CKD, *n* (%)						< 0.0001
No	3197 (86.83)	681 (77.47)	800 (85.96)	847 (89.14)	869 (92.89)	
Yes	718 (12.14)	296 (21.08)	181 (12.98)	147 (10.09)	94 (6.23)	
Missing	37 (1.02)	15 (1.45)	8 (1.06)	5 (0.77)	9 (0.89)	
CVD, *n* (%)						< 0.0001
No	3423 (90.85)	797 (85.26)	853 (90.93)	875 (91.66)	898 (94.46)	
Yes	528 (9.12)	195 (14.74)	136 (9.07)	124 (8.34)	73 (5.43)	
Missing	1 (0.03)	0 (0.00)	0 (0.00)	0 (0.00)	1 (0.10)	
Asthma, *n* (%)						0.81
No	3530 (88.22)	883 (88.81)	869 (87.31)	905 (88.95)	873 (87.87)	
Yes	422 (11.78)	109 (11.19)	120 (12.69)	94 (11.05)	99 (12.13)	

Abbreviations: BMI, body mass index; CKD, chronic kidney disease; CVD, cardiovascular disease; DM, diabetes mellitus; eGFR, estimated glomerular filtration rate; HDL‐C, high‐density lipoprotein cholesterol; LCR, lymphocyte‐to‐C‐reactive protein ratio; PIR, poverty income ratio; SE, standard error; TC, total cholesterol.

The clinical and biochemical characteristics of participants stratified by ED status are summarized in S1 Table. Compared with non‐ED participants, those with ED had lower TC, HDL‐C, and eGFR levels (all *p* < 0.001, weighted *t*‐tests), and were more likely to be older, Non‐Hispanic White, married or living separately, to have less than high school education, PIR ≤ 3, BMI ≥ 25 kg/m², and comorbidities, such as DM, hypertension, hyperlipidemia, CKD, and CVD. They were also less likely to have asthma (*p* = 0.04, Chi‐square test). All differences, unless otherwise specified, were statistically significant at *p* < 0.05.

### 3.2 Association between LCR and ED Risk

As depicted in Table 2, our univariate analysis identified that age ≥ 50, having a BMI ≥ 25 kg/m^2^, living separately, former smoking, prior alcohol consumption, and prior history of DM, hypertension, hyperlipidemia, CKD and CVD were all directly linked to ED (*p* < 0.05). Alternately, LCR, never married, having an education status higher than HS graduation, a family PIR > 3, a moderate to heavy alcohol usage, augmented eGFR levels, and a history of asthma, were inversely linked to ED (*p* < 0.01).

**Table 2:** Univariate logistic regression analysis of various variables.

Variables	OR(95% CI)	*p*‐value
Age (vs. < 50, years)		
≥ 50	11.93 (10.01,14.21)	< 0.0001
Race (vs. Mexican American)		
Non‐Hispanic Black	1.01 (0.74,1.37)	0.95
Non‐Hispanic White	1.20 (0.89,1.63)	0.21
Other race	1.15 (0.67,1.98)	0.60
BMI (vs. < 25, kg/m^2^)		
≥ 25	1.37 (1.09,1.72)	0.01
Marital status (vs. Married)		
Live separated	1.40 (1.06,1.84)	0.02
Never married	0.29 (0.22,0.40)	< 0.0001
Missing	0.00 (0.00,0.00)	< 0.0001
Education level (vs. Less than high school)		
High school	0.29 (0.22,0.40)	< 0.0001
More than high school	0.23 (0.18,0.31)	< 0.0001
Missing	0.00 (0.00,0.00)	< 0.0001
Family PIR (vs. < 1)		
1–3	1.25 (0.87,1.79)	0.22
> 3	0.72 (0.55,0.93)	0.02
Smoking status (vs. Never)		
Former	2.91 (2.42, 3.50)	< 0.0001
Now	1.16 (0.93, 1.45)	0.18
Missing	41.11 (3.88,435.24)	0.003
Alcohol usage (vs. Never)		
Former	2.22 (1.43,3.45)	< 0.001
Moderate	0.30 (0.09,1.03)	0.06
Heavy	0.52 (0.34,0.78)	0.003
Missing	0.97 (0.62,1.51)	0.88
DM (vs. No)		
Yes	5.59 (4.32,7.24)	< 0.0001
Hypertension (vs. No)		
Yes	3.58 (3.08,4.17)	< 0.0001
Missing	8.70 (0.63,120.22)	0.10
Hyperlipidemia (vs. No)		
Yes	1.68 (1.43,1.96)	< 0.0001
CKD (vs. No)		
Yes	6.46 (5.40,7.74)	< 0.0001
Missing	1.06 (0.35,3.17)	0.92
CVD (vs. No)		
Yes	6.64 (5.25,8.40)	< 0.0001
Asthma (vs. No)		
Yes	0.77 (0.60,0.99)	0.04
LCR continuous	0.90 (0.84,0.97)	0.01
TC (mmol/L)	0.92 (0.84,1.00)	0.06
HDL‐C (mmol/L)	0.91 (0.70,1.17)	0.44
eGFR (ml/min/1.73m^2^)	0.96 (0.95,0.96)	< 0.0001

Abbreviations: BMI, body mass index; CI, confidence interval; CKD, chronic kidney disease; CVD, cardiovascular disease; DM, diabetes mellitus; eGFR, estimated glomerular filtration rate; HDL‐C, high‐density lipoprotein cholesterol; LCR, lymphocyte‐to‐C‐reactive protein ratio; OR, odds ratio; PIR, poverty income ratio; TC, total cholesterol.

The multivariate analysis (Table 3) indicated robust connections between exposure and endpoint variables, which remained following confounder adjustments (*p‐*value < 0.05). Upon treatment as a continuous variable, a markedly negative correlation was evident between LCR and ED risk in the **crude model** (odds ratio [OR] = 0.90, 95% confidence interval [CI]: 0.84–0.97, *p* = 0.01). Upon treatment as categorical variables (separated into quartiles), the largest LCR quartile was negatively associated with ED in relation to the minimum quartile in adjusted **model 2** (OR = 0.63, 95% CI: 0.41–0.95, *p* = 0.03). Moreover, participants in the largest quartile also exhibited a markedly decreased ED risk by 37% compared to those with the least LCR quartile. The associated *P*‐value across all quartiles was 0.02 in the adjusted **model 2**.

**Table 3:** Relationship between LCR and ED, showed by weighted multivariate logistic regression.

	Crude model		Model 1		Model 2	
Erectile dysfunction	OR(95% CI)	*p*‐value	OR(95% CI)	*p*‐value	OR(95% CI)	*p*‐value
LCR continuous	0.90(0.84,0.97)	0.01	0.97(0.91, 1.02)	0.23	1.01(0.98, 1.04)	0.61
LCR categories						
Q1	0.55(0.43,0.71)	< 0.0001	0.55(0.41, 0.73)	< 0.001	0.64(0.41, 1.00)	0.05
Q2	0.47(0.36,0.61)	< 0.0001	0.54(0.41, 0.71)	< 0.001	0.56(0.35, 0.90)	0.02
Q3	0.26(0.21,0.32)	< 0.0001	0.36(0.29, 0.46)	< 0.0001	0.63(0.41, 0.95)	0.03
Q4		< 0.0001		< 0.0001		0.02
*P* for trend		< 0.0001		< 0.0001		0.02

Abbreviations: CI, confidence interval; OR, odds ratio; Ref, reference.

**Crude model:** LCR (lymphocyte‐to‐C‐reactive protein ratio). **Model 1:** LCR, age. **Model 2:** LCR, age, BMI (body mass index), marital status, education level, family PIR (poverty income ratio), smoking status, DM (diabetes mellitus), alcohol consumption, hypertension, hyperlipidemia, CVD (cardiovascular disease), CKD (chronic kidney disease), asthma, eGFR (estimated glomerular filtration rate).

**Figure 2:**
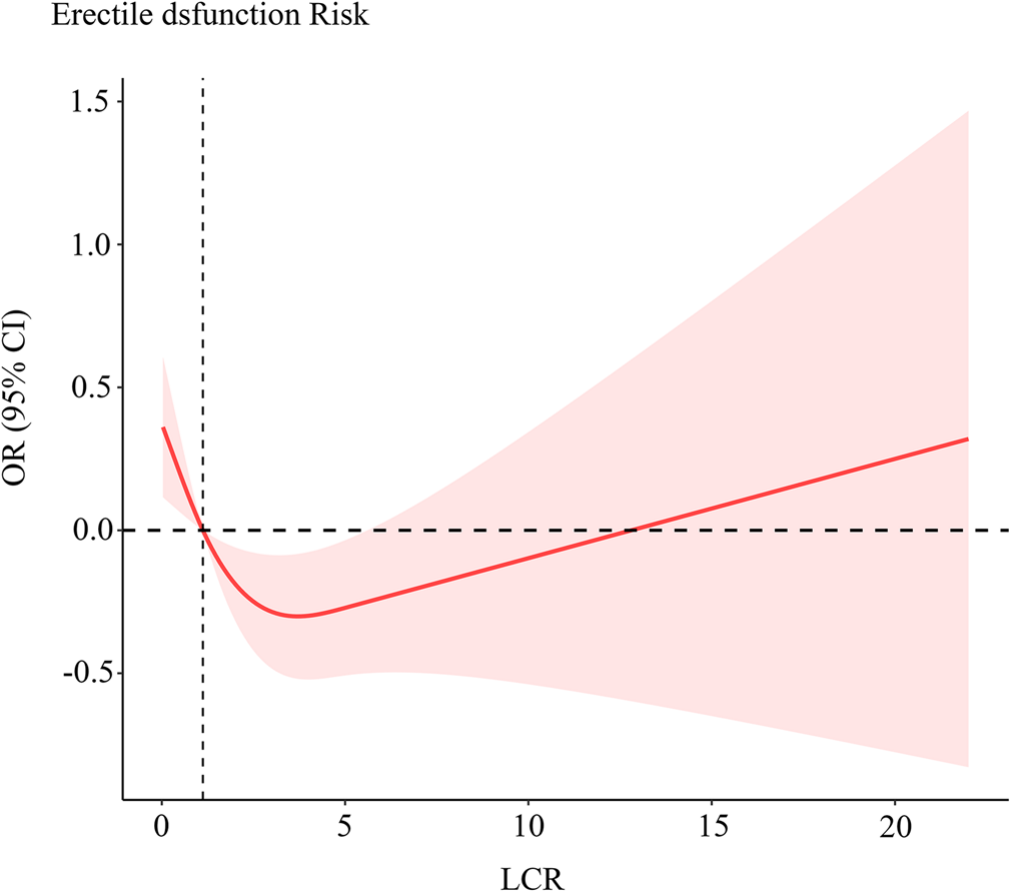
Restricted cubic spline analysis of the association between LCR values and ED risk.

After adjusting for possible covariates, we observed a **U‐shaped** link between LCR and ED (Figure 2). Therefore, we conducted a two‐piece‐wise regression model analysis and confirmed the superiority of this model in relation to the nonlinear model in elucidating the critical relationship between LCR and ED (*P* for log‐likelihood ratio < 0.005) (Table 4). The critical LCR value was 3.67, with LCR ranging between 0 and 3.67, a 1 unit rise in LCR indicated a 0.31 decline in ED (OR = 0.70, 95% CI: 0.51–0.94, *p* = 0.01). Nevertheless, at LCR range > 3.67, the aforementioned association between LCR and ED was nonexistent (OR = 1.00, 95% CI: 0.97–1.03, *p* = 0.87).

**Table 4:** Results of binary logistic regression and piecewise linear regression model.

Outcome: erectile dysfunction	Adjusted OR (95%CI)	*p*–value
Fitting by binary logistic regression model	1.01(0.98, 1.04)	0.61
Fitting by the two–piecewise linear model		
Inflection point	3.67	
LCR < 3.67	0.70(0.51, 0.94)	0.01
LCR ≥ 3.67	1.00(0.97, 1.03)	0.87
Log–likelihood ratio	0.001	

Age, race, education level, BMI (body mass index), marital status, family PIR (body mass index), smoking status, alcohol use, DM (diabetes mellitus), hypertension, hyperlipidemia, CKD (chronic kidney disease), CVD (cardiovascular disease), asthma, TC (total cholesterol), HDL‐C (high‐density lipoprotein cholesterol) and eGFR (estimated glomerular filtration rate) were all adjusted. OR, odds ratio; CI, confidence interval; Ref, reference.

All models were adjusted for age, education level, BMI (body mass index), marital status, family PIR (body mass index), smoking status, alcohol use, DM (diabetes mellitus), hypertension, hyperlipidemia, CKD (chronic kidney disease), CVD (cardiovascular disease), asthma and eGFR (estimated glomerular filtration rate). OR, odds ratio; CI, confidence interval;

### 3.3 Stratified Analyses

As depicted in Table 5, we next performed subgroup assessment based on several confounding factors. Across all subcohorts, subjects in quartile 4 persistently exhibited lower ED risks relative to subjects in quartile 1 (all OR < 1), indicating that the negative link between LCR quartiles and the risk of ED is robust.

**Table 5:** Subgroup analyses of the relationship between LCR values and the prevalence of ED.

Characteristics	Q1	Q2OR(95% CI)	Q3OR(95% CI)	Q4OR(95% CI)	*p* for trend	*p* for interaction
Age (years)						0.3
< 50	ref	0.44 (0.24,0.78)	0.66 (0.35,1.24)	0.43 (0.28,0.65)	0.01	
≥ 50	ref	0.58 (0.41,0.83)	0.50 (0.36,0.69)	0.33 (0.26,0.44)	< 0.0001	
Race						0.32
Mexican American	ref	0.57 (0.28,1.14)	0.51 (0.33,0.77)	0.38 (0.20,0.73)	0.01	
Non‐Hispanic Black	ref	0.59 (0.35,1.01)	0.48 (0.26,0.88)	0.34 (0.21,0.55)	< 0.001	
Non‐Hispanic White	ref	0.51 (0.37,0.71)	0.44 (0.32,0.60)	0.21 (0.17,0.28)	< 0.0001	
Other race	ref	1.09 (0.41,2.90)	0.76 (0.22,2.59)	0.60 (0.22,1.64)	0.19	
Education level						0.06
Less than high school	ref	0.54 (0.26,1.13)	0.61 (0.32,1.14)	0.31 (0.15,0.66)	0.01	
High school	ref	0.74 (0.52,1.03)	0.63 (0.43,0.90)	0.40 (0.27,0.60)	< 0.0001	
More than high school	ref	0.47 (0.34,0.64)	0.37 (0.27,0.50)	0.18 (0.12,0.27)	< 0.0001	
BMI (kg/m^2^)						0.88
< 25	ref	0.59 (0.38,0.90)	0.44 (0.30,0.65)	0.28 (0.20,0.39)	< 0.0001	
≥ 25	ref	0.56 (0.40,0.76)	0.48 (0.37,0.64)	0.27 (0.20,0.35)	< 0.0001	
Smoking status		0.56 (0.40,0.77)				0.57
Never	ref	0.56 (0.40,0.78)	0.56 (0.38,0.82)	0.26 (0.17,0.40)	< 0.0001	
Former	ref	0.56 (0.40,0.79)	0.39 (0.26,0.60)	0.21 (0.14,0.31)	< 0.0001	
Now	ref	0.56 (0.40,0.80)	0.49 (0.23,1.02)	0.41 (0.23,0.71)	0.004	
Family PIR		0.56 (0.40,0.81)				0.18
< 1	ref	0.56 (0.40,0.82)	0.85 (0.45,1.62)	0.39 (0.16,0.95)	0.05	
1–3	ref	0.56 (0.40,0.83)	0.49 (0.33,0.72)	0.32 (0.22,0.47)	< 0.0001	
> 3	ref	0.56 (0.40,0.84)	0.40 (0.28,0.57)	0.17 (0.12,0.25)	< 0.0001	
DM		0.56 (0.40,0.85)				0.23
No	ref	0.56 (0.40,0.86)	0.46 (0.35,0.61)	0.28 (0.22,0.37)	< 0.0001	
Yes	ref	0.56 (0.40,0.87)	0.88 (0.43,1.78)	0.48 (0.24,0.95)	0.13	
Hypertension		0.56 (0.40,0.88)				0.96
No	ref	0.56 (0.40,0.89)	0.52 (0.34,0.79)	0.33 (0.24,0.46)	< 0.0001	
Yes	ref	0.56 (0.40,0.90)	0.51 (0.38,0.68)	0.32 (0.22,0.45)	< 0.0001	
Hyperlipidemia		0.56 (0.40,0.91)				0.06
No	ref	0.56 (0.40,0.92)	0.29 (0.19,0.44)	0.21 (0.14,0.32)	< 0.0001	
Yes	ref	0.56 (0.40,0.93)	0.53 (0.40,0.72)	0.30 (0.22,0.40)	< 0.0001	
CKD		0.56 (0.40,0.94)				0.07
No	ref	0.56 (0.40,0.95)	0.47 (0.35,0.64)	0.30 (0.21,0.41)	< 0.0001	
Yes	ref	0.56 (0.40,0.96)	0.99 (0.52,1.89)	0.49 (0.27,0.88)	0.14	
CVD		0.56 (0.40,0.97)				0.29
No	ref	0.56 (0.40,0.98)	0.46 (0.34,0.62)	0.26 (0.20,0.34)	< 0.0001	
Yes	ref	0.56 (0.40,0.99)	0.84 (0.43,1.65)	0.47 (0.23,0.95)	0.08	
Asthma		0.56 (0.40,0.100)				0.1
No	ref	0.56 (0.40,0.101)	0.49 (0.38,0.63)	0.28 (0.22,0.34)	< 0.0001	
Yes	ref	0.56 (0.40,0.102)	0.25 (0.09,0.73)	0.12 (0.04,0.33)	< 0.001	

Abbreviations: BMI, body mass index; CI, confidence interval; CKD, chronic kidney disease; CVD, cardiovascular disease; DM, diabetes mellitus; LCR, lymphocyte‐to‐C‐reactive protein ratio; OR, odds ratio; PIR, poverty income ratio; Ref, reference.

Additionally, we evaluated relationships with age, ethnicity, educational level, BMI, smoking, family PIR, DM, hypertension, hyperlipidemia, CKD, CVD and asthma to elucidate strong dependency of the effect modulator on this correlation. Herein, we did not identify any significant *P‐*based association, suggesting a lack of heavy reliance on effect modifiers to mediate this relationship (all *P* for interaction > 0.05).

## 4 Discussion

This observational investigation evaluated a standardized data set from a purely US population. We found that participants with higher LCR index independently associated with decreased likelihood of ED. Following potential variable adjustment, we revealed a significant link between augmented LCR and reduced ED risk. In addition, this correlation remained substantial even in subcohort analyses. We postulate that the combination use of lymphocyte and CRP can detect high‐risk groups prone to ED earlier. Thus, early strategies can be put in place to minimize ED development risk.

With rising ED incidence, the erectile function of men demands extra research and analyses. Based on extensive surveys, males of advanced age, who suffer from ED, also exhibit additional Comorbidities, namely, CVD, diabetes, obesity, and lower urinary symptoms, which are potential ED RFs [2, 22]. Herein, we demonstrated a substantial increase in CVD incidence among the ED versus control cohort. Endothelial damage is critical to ED and CVD etiologies [23, 24, 25]. Emerging evidence suggest that ED pathogenesis and severity is closely associated with a rise in inflammatory markers. Moreover, a low‐grade subclinical inflammation can potentially influence endothelial function and eventually cause thrombosis. Inflammatory mediators, such as, interleukin (IL)‐1b, TNF‐a, IL‐6, CRP, IL‐10, as well as biomarkers are strongly upregulated among ED patients [26, 27]. LCR is a newly discovered inflammatory biomarker with robust activities affecting multiple health processes. Yoshinaga et al. [11] reported that LCR and lymphocyte CRP score are potent nutrition‐inflammation bioindicators of gastric cancer patient prognosis. Chen et al. [10] revealed that baseline LCR is a stand‐alone prognostic indicator among patients receiving hemodialysis. Considering these evidence, herein, we examined the association between ED and LCR within a large cohort of US adult men. Our analyses suggested that the LCR levels among ED sufferers were substantially reduced, relative to controls. Moreover, there existed a strong inverse relation between LCR (< 3.67, inflection point) and ED risk.

Prior investigations reported links between ED and multiple RFs, namely, BMI, smoking habit, hypertension, DM, CVD, and certain inflammatory indexes, such as, leukocytes and CRP [28, 29]. Currently, the LCR index, which integrates data from routine blood lymphocytes and CRP, has become a potential and dependable indicator of inflammatory status [30]. Herein, using univariate analysis, we demonstrated that the patient age, BMI, DM, living separately, CKD, CVD, hyperlipidemia and hypertension factors were strong RFs for ED risk. In subgroup analysis, we revealed that low‐income and reduced education status (below high school or high school) were significant factors influencing ED risk. One prior study proposed a close correlation between low‐income status and ED risk [31]. Analysis of the NHSLS data also resulted in comparable conclusions; i.e., they reported that a reduced education status equated enhanced ED incidence, however, this correlation failed to achieve significance. The aforementioned study did not adjust for comorbidities and lifestyle RFs [32]. Thus, further exploration was necessary involving a more comprehensive analyses of the ED and educational status and occupation link. Following proper adjustment of all RFs, only occupation revealed a marked correlation with ED incidence, with augmented ED cases among blue‐ versus white‐collar men [33]. Regrettably, the aforementioned study did not assess income‐based influence. According to MARSH, augmented education status was indeed correlated with reduced ED suffering [34].

Apart from the highly reported link between ED and cardiovascular RFs, namely, obesity, hypertension, hyperlipidemia, CKD, smoking, and diabetes, multiple scientists also reported a strong association between ED and CVD [29, 35]. Therefore, ED may be a potential early indicator for CVD development. The aforementioned RFs typically promote endothelial dysfunction, which ultimately result in atherosclerosis. Atherosclerosis affects all vessels in a similar manner, however, the symptomology that emerges differs based on the impacted artery diameter [36, 37, 38]. Penile arteries are smaller in diameter (1–2 mm) than coronary arteries (3–4 mm), therefore, equal severity of endothelial dysfunction and atherosclerosis can potentially induce a marked reduction in blood flow to the penis [33, 35]. It was proposed that atherosclerosis formation is an active inflammatory condition rather than a passive outcome of lipid infiltration causing damage to the blood vessels [39, 40]. Multiple clinical trials revealed the importance of inflammation in atherosclerosis pathogenesis and progression, as well as its ability to transform stable atherosclerotic lesions to unstable plaques [39]. Subclinical inflammation can negatively influence endothelial function and accelerate thrombotic events. Given these evidence, inflammation is a critical potential component of ED progression. Multiple studies examined the association of LCR with CVD development. Being a newly discovered inflammatory marker, Liu et al. [12] proposed the usage of preoperative LCR as a potent prognostic indicator of major adverse cardiovascular events (MACEs) alongside prolonged outcomes among ST‐segment elevation myocardial infarction (STEMI) patients who receive primary percutaneous coronary treatment.

A healthy vascular endothelium generally resists inflammatory invasion. However, in presence of augmented inflammation and oxidative stress (OS), the endothelial function becomes compromised [41]. It was further reported that excess inflammation induces acute or chronic arterial functional damage [42, 43, 44, 45]. Of note, circulating CRP content are substantially enhanced among ED patients, relative to their age‐ and coronary risk score‐matched non‐ED counterparts [27]. Moreover, among ED sufferers with no CVD diagnosis, the CRP content is intricately linked to penile artery disease severity, as detected via penile Doppler ultrasonography [46]. One study reported strongly upregulated fibrinogen levels among ED versus non‐ED individuals [47]. Additionally, several investigations suggested a critical association between augmented inflammation and ED among obesity syndrome or metabolic disorders sufferers [48, 49]. Nonetheless, it is important to note that, despite the robust conclusions of the aforementioned investigations, a causal association is lacking. Although penile vessels may succumb to excess inflammation from other areas of the body, this organ itself is also capable of producing substantial inflammation. The male corpus cavernosum serves a paracrine function by synthesizing angiotensin II [50], and polymorphisms within its gene is prevalent among male organic ED sufferers [51]. Angiotensin II aggravates inflammation within vessels by enhancing OS, which modulates recruitment of inflammatory factors like IL‐6 [52]. Additionally, it increases adhesion molecule production to upregulate monocytes/macrophages invasion into the vessel wall [53]. Till date, multiple investigations have hinted toward a strong correlation between inflammation and ED. However, this association is complicated, and the causal and pathological signaling networks needs further elucidation.

Herein, we demonstrated a strong inverse relation between LCR and ED, which is suggestive of a protective role of lymphocyte count against ED. Lymphocytes are critical immune cells in that they modulate the inflammatory response. In general, inflammation activates lymphocyte proliferation and activity, which, in turn, boosts lymphocyte count [54]. Conversely, reduced lymphocyte count is linked to immune cell apoptosis and functional impairment [55]. Hence, LCR is a rather simple, new, cost‐effective, and easily detectable inflammatory feature with enhanced sensitivity and minimal specificity. Among its abilities is the detection of alterations in LCR levels well before symptoms emerge. This allows LCR to be a potent early indicator of an continuing pathological condition. LCR is also an indicator of immune cell activation and systemic inflammation, which can have wide‐ranging application in clinical medicine [56]. In this investigation, we demonstrated a strong link between LCR status and ED, therefore, opening up the possibility of LCR index application in ED patient screening as well.

This study has several notable strengths. First, data was acquired from NHANES, and all assessments involved recommended NHANES sample weight adjustments. Second, we conducted careful adjustment of confounding factors to enhance reliability of our findings and increase generalizability to a broader patient population. Third, owing to its relatively low cost, easy approach, and wide‐ranging informative parameters, routine blood and blood biochemical evaluation unlocks significant potential for nephrolithiasis diagnosis and management. As a result, this screening method requires addition exploration and detailed analyses.

Regardless, this study also had its limitations. Firstly, data on ED presence or absence was acquired from a questionnaire, which may have introduced certain recall, recording, and interviewer bias. Secondly, the clinical reports in NHANES lacked details, for example, prior drug consumption or ED classification, which are areas that require critical research. Additionally, blood samples were obtained from a single blood draw. Sequential examination is a better representative of blood biology due to the unique characteristic of the blood cell life cycle. Thirdly, the NHANES database has inherent limitations, thus, it is possible that unevaluated and/or unknown confounding factors may have affected our results. Fourthly, owing to the random nature of missing information in the database, as well as the large sample population, we did not conduct multiple imputation techniques to manage missing data, which may have impacted our conclusions. Fifthly, physical activity, a relevant factor potentially influencing both systemic inflammation and erectile function, was not incorporated as a covariate in our analysis. Although NHANES collected self‐reported physical activity data, variability in reporting and absence of standardized intensity/duration thresholds limited its inclusion. Future studies should incorporate objective and detailed physical activity metrics to better understand its moderating role in the LCR–ED association. Finally, we used a cross‐sectional research design, which likely limited our capability of determining a causal association between LCR and ED.

## 5 Conclusion

In this report, we demonstrated a strong relation between reduced LCR and elevated ED risk among US adult males. Moreover, this correlation remained even in subgroup analyses. The inverse correlation between LCR and ED risk was more prominent at LCR < 3.67. LCR may thus serve a critical function in high‐risk patient screening and subsequent establishment of timely and personalized interventions. Nevertheless, additional large‐scale prospective studies are needed for verification.

## Author Contributions

**Junjie Ni:** conceptualization, writing – original draft, data curation, methodology, formal analysis, visualization, **Cong Jin:** writing – review and editing, data curation, formal analysis, **Chaoyang Xu:** writing – review and editing, formal analysis, data curation, **Shunlan Ni:** writing – review and editing, validation, formal analysis, funding acquisition, **Lin Lv:** writing – review and editing, conceptualization, visualization, project administration, supervision.

## Ethics Statement

The studies involving human participants were reviewed and approved by National Center for Health Statistics Research Ethics Review Board. The patients/participants provided their written informed consent to participate in this study.

## Conflicts of Interest

The authors declare no conflicts of interest.

## Transparency Statement

The lead author Lin Lv affirms that this manuscript is an honest, accurate, and transparent account of the study being reported; that no important aspects of the study have been omitted; and that any discrepancies from the study as planned (and, if relevant, registered) have been explained.

## Supporting information


**S1 Table:** Initial descriptions of participants between with and without ED from the 2001–2004 cycles.

## Data Availability

The data that support the findings of this study are openly available in National Health and Nutrition Examination Survey at https://www.cdc.gov/nchs/nhanes/.
