# Supplementary material for: Correlation of Lymphocyte‐to‐C‐Reactive Protein Ratio With Erectile Dysfunction Risk in United States Adult Males during 2001–2004: A Cross‐Sectional Analysis
Source: Health Sci Rep. 2025 Nov 26;8(12):e71585. doi: 10.1002/hsr2.71585 (PMC12657620; doi:10.1002/hsr2.71585)
Supplement: Supplementary file 1 — S1 Table: Initial descriptions of participants between with and without ED from the 2001–2004 cycles. [file HSR2-8-e71585-s001.doc]

| **S1 Table.** **Initial descriptions of participants between with and without ED from the 2001–2004 cycles** | | | | |
| --- | --- | --- | --- | --- |
| **Characteristics (weighted)** | **Erectile dysfunction** | | | |
|  | **Total (N = 3952)** | **No (N = 2794)** | **Yes (N = 1158)** | ***P*-value** |
| Age (years) | 45.17±0.38 | 41.25±0.31 | 61.41±0.48 | < 0.0001 |
| TC (mmol/L) | 5.18±0.02 | 5.20±0.03 | 5.11±0.04 | 0.05 |
| HDL-C (mmol/L) | 1.22±0.01 | 1.22±0.01 | 1.21±0.01 | 0.43 |
| eGFR (ml/min/1.73m^2^) | 93.14±0.55 | 96.24±0.59 | 80.32±0.86 | < 0.0001 |
| LCR, n (%) |  |  |  | < 0.0001 |
| Q1 | 992(21.74) | 566(18.46) | 426(35.34) |  |
| Q2 | 989(24.80) | 696(24.53) | 293(25.91) |  |
| Q3 | 999(26.23) | 723(26.80) | 276(23.87) |  |
| Q4 | 972(27.22) | 809(30.21) | 163(14.88) |  |
| Age (years, n (%)) |  |  |  | < 0.0001 |
| <50 | 2036(63.01) | 1884(73.65) | 152(18.99) |  |
| >=50 | 1916(36.99) | 910(26.35) | 1006(81.01) |  |
| Race, n (%) |  |  |  | 0.49 |
| Mexican American | 812(7.81) | 575(8.03) | 237(6.90) |  |
| Non-Hispanic Black | 720(9.32) | 547(9.56) | 173(8.29) |  |
| Non-Hispanic White | 2162(74.38) | 1479(73.89) | 683(76.42) |  |
| Other race | 258(8.49) | 193(8.51) | 65(8.40) |  |
| Marital status, n (%) |  |  |  | < 0.0001 |
| Married | 2720(69.91) | 1869(68.27) | 851(76.71) |  |
| Live separated | 550(11.54) | 319(10.39) | 231(16.30) |  |
| Never married | 680(18.44) | 604(21.21) | 76(6.99) |  |
| Missing | 2(0.11) | 2(0.14) | 0(0.00) |  |
| Education level, n (%) |  |  |  | < 0.0001 |
| Less than high school | 533(6.02) | 259(4.06) | 274(14.14) |  |
| High school | 1556(37.92) | 1125(37.78) | 431(38.52) |  |
| More than high school | 1861(55.99) | 1408(58.08) | 453(47.35) |  |
| Missing | 2(0.06) | 2(0.08) | 0(0.00) |  |
| Family PIR, n (%) |  |  |  | < 0.0001 |
| < 1 | 553( 9.83) | 374(10.15) | 179(11.11) |  |
| 1-3 | 1548(32.13) | 1013(31.52) | 535(43.14) |  |
| > 3 | 1645(53.15) | 1266(58.33) | 379(45.75) |  |
| BMI (kg/m^2^, n (%)) |  |  |  | 0.01 |
| <25 | 1144(29.04) | 854(30.67) | 290(24.41) |  |
| >=25 | 2711(69.46) | 1905(69.33) | 806(75.59) |  |
| Smoking status, n (%) |  |  |  | < 0.0001 |
| Never | 1587(42.66) | 1243(45.72) | 344(30.03) |  |
| Former | 1297(29.13) | 721(24.73) | 576(47.31) |  |
| Now | 1064(28.18) | 829(29.54) | 235(22.52) |  |
| Missing | 4(0.03) | 1(0.01) | 3(0.14) |  |
| Alcohol usage, n (%) |  |  |  | < 0.0001 |
| Never | 285(6.98) | 196(6.97) | 89(6.99) |  |
| Former | 817(16.73) | 438(13.51) | 379(30.03) |  |
| Moderate | 63(1.93) | 58(2.24) | 5(0.68) |  |
| Heavy | 944(25.33) | 796(27.96) | 148(14.45) |  |
| Missing | 1843(49.04) | 1306(49.32) | 537(47.85) |  |
| DM, n (%) |  |  |  | < 0.0001 |
| No | 3380(89.53) | 2556(93.65) | 824(72.51) |  |
| Yes | 572(10.47) | 238(6.35) | 334(27.49) |  |
| Hypertension, n (%) |  |  |  | < 0.0001 |
| No | 2466(68.15) | 1999(73.96) | 467(44.12) |  |
| Yes | 1483(31.72) | 794(25.97) | 689(55.52) |  |
| Missing | 3(0.13) | 1(0.07) | 2(0.36) |  |
| Hyperlipidemia, n (%) |  |  |  | < 0.0001 |
| No | 1086(27.97) | 835(29.85) | 251(20.23) |  |
| Yes | 2866(72.03) | 1959(70.15) | 907(79.77) |  |
| CKD, n (%) |  |  |  | < 0.0001 |
| No | 3197(86.83) | 2485(91.83) | 712(66.15) |  |
| Yes | 718(12.14) | 284(7.10) | 434(33.03) |  |
| Missing | 37(1.02) | 25(1.07) | 12(0.81) |  |
| CVD, n (%) |  |  |  | < 0.0001 |
| No | 3423(90.85) | 2608(94.96) | 815(73.84) |  |
| Yes | 528(9.12) | 186(5.04) | 342(26.01) |  |
| Missing | 1(0.03) | 0(0.00) | 1(0.14) |  |
| Asthma, n (%) |  |  |  | 0.04 |
| No | 3530(88.22) | 2478(87.72) | 1052(90.29) |  |
| Yes | 422(11.78) | 316(12.28) | 106(9.71) |  |
| Continuous data are shown as as means and SE, while categorical data are presented as percentages.  **Abbreviations:** HDL-C, high-density lipoprotein cholesterol; TC, total cholesterol; PIR, poverty income ratio; BMI, body mass index; LCR, lymphocyte-to-C-reactive protein ratio; CKD, chronic kidney disease; DM, diabetes mellitus; eGFR, estimated glomerular filtration rate; CVD, cardiovascular disease. | | | | |
